# Effect of esketamine on the EC50 of remifentanil for blunting cardiovascular responses to endotracheal intubation in female patients under general anesthesia: a sequential allocation dose-finding study

**DOI:** 10.1186/s12871-024-02454-4

**Published:** 2024-02-21

**Authors:** Fan Ziqiang, He Keyu, Xue Yun, Liu Li, Bai Yiping

**Affiliations:** 1 Southwest Medical University, Luzhou, 646000 Sichuan Province China; 2 Chongqing University Fuling Hospital, Fuling, Chongqing, 408000 China; 3 Anesthesiology and Critical Care Medicine Key Laboratory of Luzhou, Southwest Medical University, Luzhou, 646000 Sichuan Province China; 4 Department of Anesthesiology, The Affiliated Hospital of Southwest Medical University, Luzhou, China Sichuan Province 646000

**Keywords:** Esketamine, Remifentanil, Endotracheal intubation response, Half effect-site concentration

## Abstract

**Background:**

This study aimed to investigate the effect of esketamine on the dose–effect relationship between remifentanil and the cardiovascular response to endotracheal intubation during target-controlled infusion (TCI) of propofol.

**Methods:**

Patients underwent elective gynecological laparoscopic surgery under general anesthesia with endotracheal intubation, aged 18–65 years, American Society of Anesthesiologists class I or II, 18 kg/m^2^ ≤ body mass index ≤ 30 kg/m^2^, were randomly divided into the control (group C) and esketamine groups (group E). Before anesthesia induction, group E received an intravenous injection of 0.3 mg/kg of esketamine, while group C received an equal dose of physiological saline. TCI of propofol to the effect-site concentration (EC) of 3.0 μg/mL, and then TCI of remifentanil to the effect room and intravenous injection of rocuronium 0.6 mg/kg after *MOAA/S* was 0. Endotracheal intubation was performed after 2 min. Dixon’s modified sequential method was used, and the initial EC of remifentanil was 3.0 ng/mL. The EC of remifentanil was determined according to the intubation response of the previous patient, with an adjacent concentration gradient of 0.3 ng/mL. The EC_50_ and EC_95_ values and their 95% confidence intervals (CIs) were determined using probit regression analysis.

**Results:**

The EC_50_ for cardiovascular response inhibition to endotracheal intubation using remifentanil was 3.91 ng/mL (95% CI: 3.59–4.33 ng/mL) and EC_95_ was 4.66 ng/mL (95% CI: 4.27–6.23 ng/mL) with TCI of propofol 3.0 μg/mL. After intravenous administration of 0.3 mg/kg of esketamine, the EC_50_ of remifentanil was 3.56 ng/mL (95% CI: 3.22–3.99 ng/mL) and EC_95_ was 4.31 ng/mL (95% CI: 3.91–5.88 ng/mL).

**Conclusions:**

Combined with TCI of propofol 3.0 μg/mL for anesthesia induction, esketamine significantly reduced the EC_50_ and EC_95_ of remifentanil to inhibit the cardiovascular response to endotracheal intubation.

**Trial registration:**

The trial was registered in the Chinese Clinical Trials Registry (www.chictr.org.cn; registration number: ChiCTR2200064932; date of registration:24/10/2022).

## Background

Endotracheal intubation produces strong airway stimulation, which is no less intense than the stress produced by surgical skin incision [1]. The dramatic fluctuations in hemodynamics caused by sympathetic nerve excitation and the release of catecholamines may lead to cardiovascular accidents during induction of general anesthesia [2]. Remifentanil is a short-acting μ-receptor agonist with fast metabolism. The use of larger doses can reduce the response to endotracheal intubation and cardiovascular risk due to the induction of anesthesia and endotracheal intubation [3]. Ahonen et al. reported that induction of anesthesia with remifentanil 2 μg/kg and propofol, maintained at 0.25 or 0.5 μg/(kg·min), can provide appropriate anesthesia and allow patients to quickly recover and extubate [4]. However, large doses of remifentanil may be accompanied by adverse reactions such as coughing, bradycardia, or hypotension [5, 6].

Esketamine is an intravenous anesthetic with potent analgesic effects and act on N-methyl-D-aspartate receptors (NMDAR) and opioid and monoaminergic receptors in the brain and spinal cord to produce dose-related sedative-hypnotic, analgesic, and amnestic effects [7–9]. Esketamine does not inhibit respiration and maintains hemodynamic stability. Under sedative concentration, it can effectively maintain patient's spontaneous breathing and reduce the incidence of hypoxemia [10]. Ledowski et al. reported that the combined use of 0.5 mg/kg esketamine during general anesthesia induction can improve tracheal intubation conditions [11]. During induction of general anesthesia, esketamine can significantly reduce the dosage of opioid analgesic drugs [12], improve the analgesic effect, reduce the incidence of opioid side effects [13, 14], and have protective effects on the respiratory systems [15]. For the circulatory system, esketamine increases cardiac output in a dose-dependent manner, to some extent counteracting the inhibitory effects of other anesthetic drugs on circulation, thereby maintaining the stability of patient circulation during anesthesia induction [16, 17]. However, the excitatory effect of this cardiovascular system is not always beneficial, and esketamine are known to increase myocardial VO2 and increase MAP, which may pose certain risks when applied to patients with cardiovascular diseases such as hypertension and coronary heart disease.

Propofol combined with remifentanil is the most common target-controlled infusion (TCI) regimen for anesthesia induction [18]. However, the concentration of remifentanil required to reduce endotracheal intubation stress when combined with esketamine is unclear. This trial aimed to determine the effect of esketamine on the quantitative-effect relationship of remifentanil in suppressing the cardiovascular response to endotracheal intubation under propofol anesthesia and to provide a reference for clinical application.

## Methods

### Ethical statements and study design

This prospective, double-blind, dose-finding clinical trial was approved by the Ethics Committee of Chongqing University Fuling Hospital, Chongqing, China in 2022 (approval number: 2022CQSFLZXYYEC-057) and registered in the Chinese Clinical Trial Registry (registration number: ChiCTR2200064932). All patients signed an informed consent form before the trial.

Female patients who underwent underwent elective gynecological laparoscopic surgery under general anesthesia with endotracheal intubation between November 2022 and April 2023 were included. The following inclusion criteria were applied: patients aged 18–65 years, American Society of Anesthesiologists classification I or II, and 18 kg/m^2^ ≤ body mass index ≤ 30 kg/m^2^. The exclusion criteria were as follows: patients anticipating airway difficulties; those with a history of intolerance or allergy to experimental medications; those with a history of severe cardiovascular disease, hypertension or screening systolic blood pressure (SBP) ≥ 140 mmHg and/or diastolic BP ≥ 90 mmHg, hyperthyroidism, or asthma; and those who were pregnant or lactating.

### Introduction and anesthetic management

All patients fasted for at least 8 h, abstained from drinking for 2 h before surgery, and did not receive any preoperative medications. After admission, patients were divided into the saline control group (group C) or esketamine group (group E) using a randomized numerical table method. Electrocardiography, noninvasive blood pressure (BP), heart rate (HR), and pulse oxygen saturation were monitored for all patients (N15 Anesthesia Monitor, Mindray, China). A venous catheter of 20- or 22-gauge was inserted in one of the arms, and 6–8 mL/kg of multiple electrolytes injection was infused as a balanced salt solution for compensatory intravascular volum expansion before anesthesia induction. During surgery, infusion pathways may be increased according to the patient's actual situation if necessary. A mask oxygen dose of 6 L/min was administered for 5 min and anesthesia was induced. Patients in group E were given esketamine 0.3 mg/kg, while patients in group C were administered an equal dose of saline. Patients in both groups were treated using a TCI-I injection pump (Beijing Oriental Chengyitong Technology Co., Ltd.) for TCI of propofol with an effect-site concentration (EC) of 3.0 μg/mL (Marsh model), followed by TCI of remifentanil (20A03171, Yichang Humanwell Pharmaceutical Co., Ltd., Minto model) at a Modified Observer's Assessment of Alertness/Sedation (MOAA/S: No response after painful trapezius squeeze is 0. Responds only after painful trapezius squeeze is 1. Responds only after mild prodding or shaking is 2. Responds only after loud and/or repeated demand is 3. Lethargic response to demand in normal tone is 4. Responds readily to demand in normal tone is 5) of 0 [19, 20]. Rocuronium 0.6 mg/kg was administered after stabilizing the drug concentration. After 2 min, a skilled anesthesiologist judged the loss of blinking reflex and performed tracheal intubation under the guidance of a videolaryngoscopy, and the procedure was completed within 30 s.

During anesthesia induction, if the MAP was < 60 mmHg or 30% below the preoperative basal value, 10 mg of ephedrine was administered intravenously. For HR < 50 beats/min, atropine 0.3 mg was given intravenously. Patients with HR < 50 beats per minute or SBP < 80 mmHg were recorded as adverse events after symptomatic treatment, and their data were not included in the calculation of drug EC_50_ and EC_95_, as the changes in blood pressure and heart rate after symptomatic treatment cannot be distinguished as being caused by drug factors or tracheal intubation stimulation. Patients in whom the first endotracheal intubation failed or those who were intubated for more than 30 s were excluded. Participants who did not complete the study or violated the study protocol were also excluded. Due to prolonged or repeated tracheal intubation, there is greater stimulation and more significant changes in heart rate and blood pressure compared to a single smooth tracheal intubation.

### Dixon’s up-and-down and sample size

The EC_50_ of remifentanil was calculated using a modified Dixon up-and-down method. The initial EC of remifentanil was set at 3.0 ng/mL with a concentration gradient of 0.3 ng/mL. If there was a positive response to endotracheal intubation, the target concentration was adjusted upward by one gradient; otherwise, the concentration was decreased by one gradient. According to the simulation study conducted by Stylianou and Flournoy, it is preliminarily estimated that the sample size of 20–40 patients can provide a stable target dose [21]. After obtaining 6 inflection points from positive to negative endotracheal intubation reactions, patient recruitment was terminated. A positive response to endotracheal intubation was defined as SBP 15% above the basal level, HR 15% above the basal level, or the presence of a lacrimal response within 2 min of endotracheal intubation [22].

### Blinding

All patients were anesthetized by one investigator, and another investigator assessed them for the presence of a positive response to endotracheal intubation. Neither the anesthesiologist performing endotracheal intubation nor the patient was aware of the anesthesia medication regimen.

### Statistical analysis

All statistical analyses were performed using SPSS 21.0 software. The Shapiro–Wilk test was used to assess the normality of continuous variables. Normally distributed measurements were expressed as mean ± standard deviation (*x* ± *S*) using an independent samples t-test, and counts were expressed as cases (%) using Fisher’s exact test. EC_50_, EC_95_, and the corresponding 95% confidence intervals (CIs) of the drugs were calculated using probit regression analysis. An independent sample t-test was used for comparison between groups of remifentanil EC_50_ and EC_95_. A two-sided *P*< 0.05 was considered statistically significant.

## Results

### Basic information

According to Dixon's sequential design requirements, recruitment of patients should be stopped when all groups reach the intersection point of positive and negative results in the 6th endotracheal intubation test. Overall, 42 female patients who underwent elective gynecological laparoscopic surgery under general anesthesia with endotracheal intubation were recruited for this study: 22 in group C and 18 in group E. The difference in the general condition of the patients in the two groups was not statistically significant (*P* > 0.05) (Table 1). There was no statistically significant difference in the general condition of patients between the positive and negative endotracheal intubation groups (*P* > 0.05) (Table 2).

**Table 1:** Comparison of preoperative general conditions between groups E and C

	Group E(*n* = 18)	Group C(*n* = 21)	***P***
ASA(I /II)	7/11	8/13	1.000
Mallampati(I /II)	8/10	13/8	0.343
Age(years)	41.44 ± 9.38	44.05 ± 9.47	0.396
BMI(kg/m^2^)	23.55 ± 2.31	23.14 ± 2.88	0.629
SBP	111.39 ± 9.59	113.81 ± 9.56	0.436
DBP	69.00 ± 8.37	70.71 ± 7.25	0.497
MAP	83.13 ± 7.98	85.08 ± 7.61	0.440
HR	74.83 ± 10.27	77.29 ± 9.81	0.451

*ASA* American Society of Anesthesiologists, *BMI* Body mass index, *SBP* Systolic blood pressure, *DBP* Diastolic blood pressure, *MAP* Mean arterial pressure, *HR* Heart rate

**Table 2:** Comparison of general conditions of patients with positive and negative reactions to endotracheal intubation

	Group E			Group C		
	Negative(*n* = 8)	Positive(*n* = 10)	***P***	Negative(*n* = 9)	Positive(*n* = 11)	***P***
Age(years)	40.38 ± 11.17	42.30 ± 8.21	0.679	44.67 ± 10.36	43.58 ± 9.19	0.803
BMI(kg/m^2^)	22.98 ± 2.34	24.01 ± 2.30	0.360	23.55 ± 3.51	22.83 ± 2.41	0.581
SBP	109.88 ± 9.03	112.60 ± 10.33	0.565	110.44 ± 10.53	116.33 ± 8.34	0.168
DBP	70.88 ± 10.56	67.50 ± 6.31	0.412	68.33 ± 7.57	72.50 ± 6.76	0.200
MAP	83.87 ± 9.35	82.53 ± 7.17	0.734	82.37 ± 7.96	87.11 ± 6.98	0.163
HR	75.13 ± 12.39	74.60 ± 8.92	0.918	72.89 ± 8.81	80.58 ± 9.54	0.074

*BMI* Body mass index, *SBP* Systolic blood pressure, *DBP* Diastolic blood pressure, *MAP* Mean arterial pressure, *HR* Heart rate

### ***EC***_***50***_*** and EC***_***95***_*** of remifentanil***

**Fig. 1:**
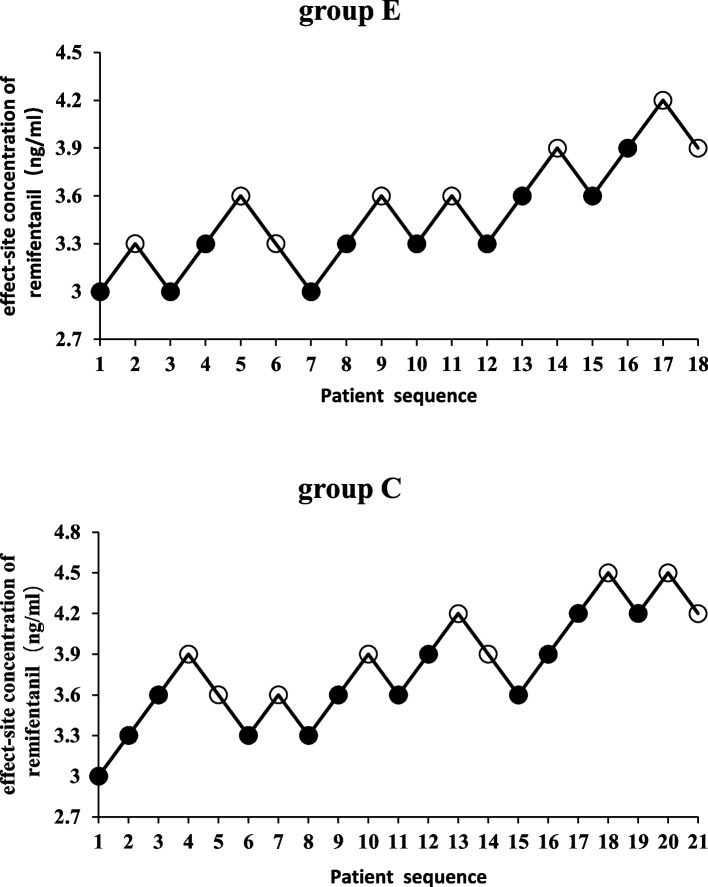
Individual cardiovascular response of endotracheal intubation (Black represents positive; white represents negative)

**Fig. 2:**
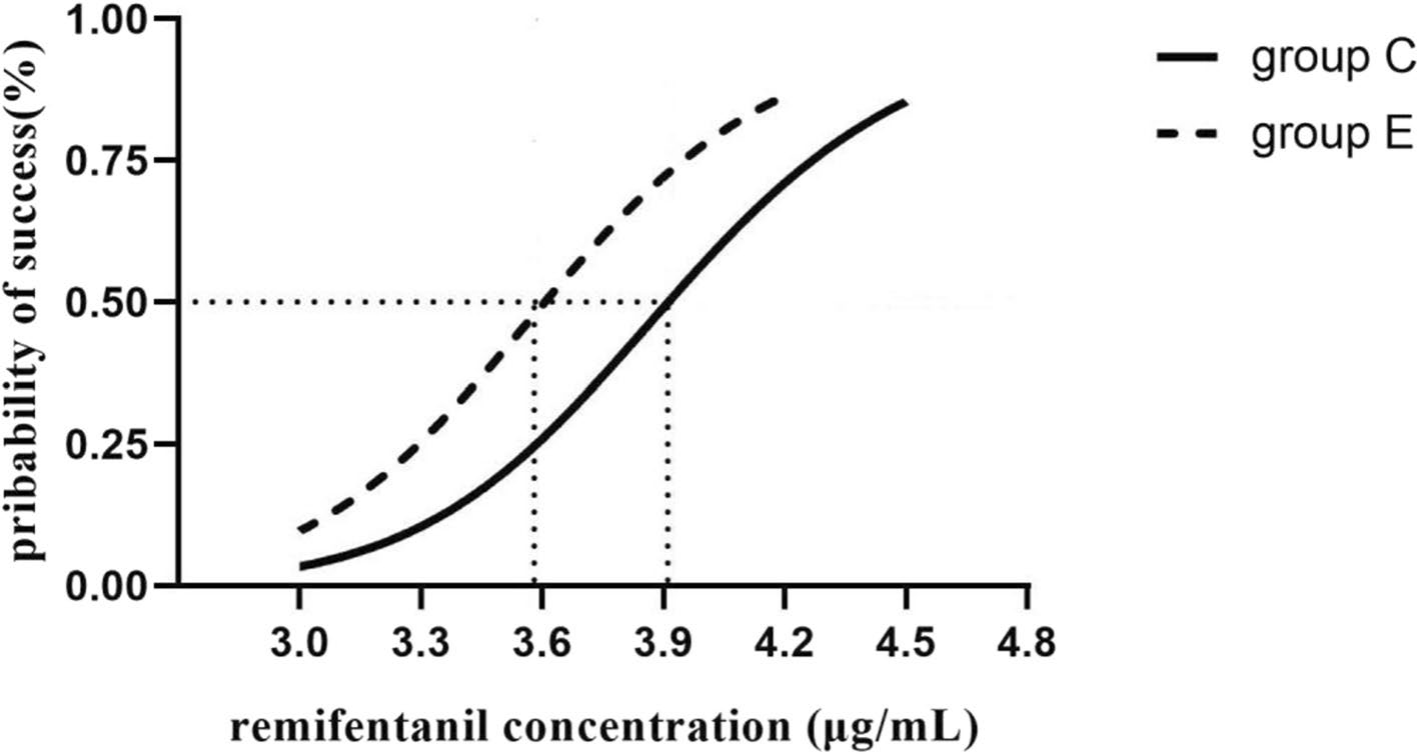
Dose–response curve of remifentanil. In group C, the EC_50_ was 3.91 ng/mL. In group E, the EC_50_ of remifentanil was 3.56 ng/mL

Sequential plots of the inhibition of cardiovascular responses to endotracheal intubation in groups E and C are shown in Fig. 1. Dose response curves are shown in Fig. 2. When propofol 3.0 μg/mL was administered by TCI, the EC_50_ of remifentanil inhibiting the cardiovascular response to endotracheal intubation in group C was 3.91 ng/mL (95% CI, 3.59–4.33 ng/mL) and the EC_95_ was 4.66 ng/mL (95% CI, 4.27–6.23 ng/mL). In group E, the EC_50_ of remifentanil was 3.56 ng/mL (95% CI, 3.22–3.99 ng/mL), and the EC95 was 4.31 ng/mL (95% CI, 3.91–5.88 ng/mL). The EC_50_ and EC_95_ values of group E were significantly lower than those of group C (*P* < 0.05).

During the induction of anesthesia, one patient in group C was intubated at a concentration of 3.6 ng/mL, and two at concentrations of 3.9 ng/mL were intubated after receiving additional remifentanil for 5 min due to delayed loss of consciousness [23]. The endotracheal intubation reaction was positive in all three patients. No adverse effects such as hypotension, bradycardia, cough, chest wall rigidity, or postoperative delirium were observed in any patient.

## Discussion

Using the improved Dixon up and down method, this study demonstrated that the EC50 of remifentanil inhibiting cardiovascular response to tracheal intubation was 3.91 ng/mL when the target controlled infusion of propofol effect chamber concentration was 3.0 µg/mL. After intravenous injection of 0.3 mg/kg esketamine, the EC_50_ decreased to 3.56 ng/mL.

As a more precise mode of drug delivery, TCI can rapidly and accurately achieve the set drug EC and maintain the desired depth of anesthesia [24]. Most previous studies have focused on the inhibition of the endotracheal intubation response at half of the plasma drug concentration; however, there are some differences between the EC and plasma drug concentration (Cp). As the plasma is not the anatomical site of action of anaesthetic drugs, a delay (hysteresis) is observed between the time course of the Cp and the drug effect [25]. The reduction in the endotracheal intubation stress response to the concentration of remifentanil when combined with esketamine remains unclear. In the present study, we determined the effect of esketamine on the quantitative relationship of remifentanil in suppressing the cardiovascular response to endotracheal intubation under propofol anesthesia.

EC_50_ is located at the midpoint of the drug’s quantitative effect curve and best reflects the potency of the drug. In this study, Dixon’s modified sequential method was used, and a small number of samples reflected the potency of the drug, suitable for the study of drugs with a rapid onset of action and short-term evaluation of the effect. The drugs used in the study were all fast-acting clinical anesthetics, and both groups had six crossings; that is, the thresholds crossed the median, which satisfied the conditions of the study design.

Remifentanil, an opioid analgesic, has been proved to effectively blunt the hemodynamic response to endotracheal intubation when administered via injection or infusion. However, the effect of small doses of remifentanil is not obvious, while large doses can cause adverse reactions such as hypotension and bradycardia [26]. Many studies have evaluated the efficacy of remifentanil in TCI for the cardiovascular response to endotracheal intubation. Mustola and Toivonen proposed that the EC_50_ and EC_95_ values for remifentanil are 3.17 ng/mL and 3.79 ng/mL, respectively, when propofol is administered to maintain a BIS value between 40 and 60 in patients [27]. Kim et al. used Ce of propofol 3.5 µg/mL for laryngeal mask airway (LMA) insertion requiring EC_50_ of remifentanil 3.04 ± 0.49 ng/mL [28]. The majority of the aforementioned research centers on identifying the necessary effect chamber concentration of propofol-induced remifentanil for suppressing related stress responses. Nevertheless, there remains a scarcity of studies on esketamine. Consequently, we can only tentatively establish an initial effective chamber concentration of remifentanil at 3.0 ng/mL, relying solely on the available research reports.

In this study, TCI of propofol 3.0 μg/mL for anesthesia induction combined with esketamine can significantly reduce the EC_50_ and EC_95_ of endotracheal intubation cardiovascular response inhibition by remifentanil. The combined use of esketamine successfully decreased the required dosage of remifentanil, potentially mitigating its side effects, although this study did not explicitly evaluate this aspect. The primary objective of this investigation was not to assess the incidence of side effects, hence the study's sample size was insufficient to produce statistically significant results regarding this matter. This may be because esketamine can act on NMDAR and opioid receptors to produce analgesia together with remifentanil [29], which reduces the intensity of patients’ stress response to endotracheal intubation and better maintains hemodynamic stability during anesthesia-induced endotracheal intubation and extubation [16]. It is widely recognized that the mitigation of tracheal intubation stress response heavily relies on analgesic agents. When esketamine is administered in combination with remifentanil, the required concentration of remifentanil to suppress tracheal intubation response decreases, primarily due to the synergistic analgesic effect between these two agents. This observation also explains why the measured concentrations of remifentanil in the control group were similar to those reported by Bouillon et al. [18].

There is still controversy over the optimal PK parameter model for propofol [30–32]. Yang, X. Y. et al. suggested that the Marsh model induces sedation faster than that using the Schnider model, with no statistically significant differences in hemodynamic changes [33]. Shunsheng C et al. suggested that in the TCI of propofol in Chinese gynecological surgery patients, the accuracy of the Marsh model is higher than that of the Schnider model [34]. Hence, the Marsh model for propofol potentially offers superior advantages among the Chinese population, prompting its selection as the preferred TCI model for propofol in this study. Okuyama et al. suggested that the predicted EC of propofol alone at which 50% of patients did not move with laryngeal mask airway insertion was 3.59 μg/mL [35]. The combined use of remifentanil can reduce the required concentration of propofol, and the use of a combination of low-dose esketamine reduces the propofol dosage [18, 36]. In addition, with reference to the propofol effect chamber concentration adopted in previous studies [28, 35], the target-controlled effector room concentration of propofol was set at 3.0 μg/mL in the study.

Three patients in group C showed delayed loss of consciousness, suggesting that there may be individual differences in the synergistic sedative effect of remifentanil and propofol. To patients experiencing delayed consciousness loss, the use of remifentanil instead of propofol was based on the study's prerequisite of a propofol effect chamber concentration of 3.0 μg/mL. Remifentanil exhibits a synergistic effect with propofol, ensuring smooth induction of anesthesia in patients. During the clinical trail, the patient did indeed enter a state of anesthesia following the administration of remifentanil, and no patient was excluded for severe hypotension or bradycardia after anesthesia induction as specified in the methods section, which may be because esketamine can act on receptors, such as NMDAR, opioid, and monoaminergic, to produce sedative-hypnotic effects and reduce propofol dosage. In addition, the stimulating effect of esketamine on the circulatory system effectively reduces the occurrence of hypotension. Previous studies indicated that low doses of esketamine can produce safe and effective anesthesia [37].

None of the patients included in this study experienced significant adverse effects such as hypotension, bradycardia, or cough, probably because remifentanil itself has less effect on the circulatory system [38] and esketamine excites the sympathetic nerves, dose-dependently increases cardiac output, and better maintains intraoperative hemodynamic stabilization when applied in combination with opioids [16, 39]. Additionally, esketamine has bronchodilating and hyperventilating effects, reduces the incidence and intensity of choking in patients, improves the body’s sensitivity to CO_2_ [15], and is more suitable for use in patients at risk of respiratory complications.

This study has some limitations. In this study, the depth of anesthesia was meticulously monitored using the MOAA/S score. Intravenous muscle relaxants are administered when the patient's MOAA/S score reached 0, indicatingan absence of response to stimulation, which corresponds to a Richmond Agitation-Sedation Scale (RASS) score of -5, followed by tracheal intubation 2 min later. This stringent protocol ensured that the patient was maintained at an appropriate level of anesthesia throughout the procedure. Sang Yun Cho et al. suggested that increased effect-site concentration of propofol might reduce EC_50_ and EC_95_ of remifentanil during endotracheal intubation [3]. There is a close relationship between the response to tracheal intubation and the depth of anesthesia, and this study did not quantitatively monitor the depth of anesthesia due to insufficient funding. Ensuring anesthesia depth solely by ensuring that the patient's MOAA/s score is 0 may have some impact on the research results, which may have a certain impact on the research results. Animal and human studies have shown sex differences in opioid-induced analgesia and related adverse events [40, 41], and differences in gene expression between sexes may be involved in the regulatory mechanisms involved [42], resulting in lower sensitivity to opioids [43] and higher opioid dosage in men than that in women [44]. In addition, esketamine are known to increase myocardial VO2 and increase MAP, which may pose certain risks when applied to patients with cardiovascular diseases. Therefore, patients with cardiovascular diseases were excluded from the clinical trail. Although ketamine has a bronchodilatory effect, its application in asthma patients is somewhat controversial [45]. The focus of this study is on the concentration requirements for drug inhibition of cardiovascular response to tracheal intubation. Asthma patients have higher airway reactivity than that of non-asthma patients. Patients with hyperthyroidism have strong excitability in the nervous system, and generally, their heart rate and blood pressure fluctuate more than those of ordinary patients. The concentration of remifentanil required to suppress cardiovascular response to tracheal intubation in the above two types of patients may be higher. Therefore, they were excluded from the study. In order to exclude the influence of gender and related disease factors, this study included female patients with ASA I/II grade and aged 18–65. However, the pharmacokinetics and pharmacodynamics of the drug are also influenced by other factors such as obesity, internal environmental status, and drug interactions. Therefore, the EC_50_ of remifentanil required to inhibit the response to endotracheal intubation in male, pediatric, and elderly patients, and those with serious comorbidities need to be further investigated because of their characteristics and changes in pharmacokinetics.

## Conclusions

In conclusion, TCI of propofol 3.0 μg/mL for anesthesia induction combined with esketamine can significantly reduce the EC_50_ and EC_95_ of endotracheal intubation cardiovascular response inhibition by remifentanil. Reducing the dosage of remifentanil may reduce related drug side effects, while ensuring stable circulation during anesthesia induction in patients. This can provide some reference for anesthesia induction medication in female patients undergoing general anesthesia who require tracheal intubation.

## Data Availability

The datasets generated and analysed during the current study are available from the corresponding author on reasonable request.

## Abbreviations

TCI: Target-controlled infusion
EC: Effect-site concentration
EC_50_: 50% Effective concentration
EC_95_: 95% Effective concentration
CI: Confidence intervals
NMDAR: N-methyl-D-aspartate receptors
SBP: Systolic blood pressure
HR: Heart rate
